# Heterologous Expression of the Melon *CmVQ23* Positively Regulates Resistance to *Verticillium dahliae* in *Arabidopsis*

**DOI:** 10.3390/plants15152283

**Published:** 2026-07-26

**Authors:** Peifeng Yu, Simin Lu, Jiyang Zhou, Xianlei Wang, Xuefei Ning

**Affiliations:** Xinjiang Key Laboratory of Biological Resources and Genetics Engineering, College of Life Science and Technology, Xinjiang University, Urumqi 830046, China; yu@xju.edu.cn (P.Y.);

**Keywords:** melon, ROS, *Verticillium dahliae*, phenylpropanoid metabolism

## Abstract

*Verticillium dahliae* is a devastating soil-borne fungal pathogen that causes severe yield losses in melon (*Cucumis melo* L.) and other crops. Identifying novel resistance genes is crucial for sustainable disease management. In this study, we characterized the function of *CmVQ23*, a candidate gene previously identified through QTL mapping, in mediating defense against *V. dahliae* using heterologous expression in *Arabidopsis thaliana*. Subcellular localization assays revealed that the CmVQ23-eGFP fusion protein predominantly localized to the nucleus, consistent with its predicted role as a co-factor of transcription factor. Upon *V. dahliae* inoculation, *CmVQ23*-overexpressing *Arabidopsis* lines exhibited significantly reduced disease indices and restricted fungal proliferation compared with wild-type and mutant plants, although these lines displayed altered vegetative growth, including delayed bolting and reduced plant height. Mechanistically, *CmVQ23* overexpression promoted reactive oxygen species (ROS) accumulation and hypersensitive response (HR)-mediated cell death at infection sites, as evidenced by intensified DAB and trypan blue staining. Furthermore, transgenic lines maintained higher photosynthetic efficiency, enhanced antioxidant enzyme activities, and increased lignin deposition via upregulation of phenylalanine ammonia-lyase (PAL) and polyphenol oxidase (PPO). Notably, *CmVQ23* overexpression markedly upregulated both salicylic acid (SA)- and jasmonic acid/ethylene (JA/ET)-responsive marker genes, including *AtPR1*, *AtPR2*, *AtPR5*, *AtPAD4*, *AtPDF1.2*, and *AtVSP2* upon infection. Collectively, these findings demonstrate that CmVQ23 functions as a positive regulator of resistance to *Verticillium dahliae* by orchestrating ROS/HR-mediated cell death, antioxidant defense, phenylpropanoid pathway activation, and phytohormone signaling crosstalk, offering a promising genetic resource for improving Verticillium wilt resistance in crops.

## 1. Introduction

*Verticillium dahliae* is a soil-borne phytopathogenic fungus that causes devastating vascular wilt diseases in a broad range of economically important crops, including cotton, tomato, and melon, leading to substantial yield losses worldwide [1,2,3]. Unlike other major crops such as cotton and tomato, where considerable progress has been made in identifying resistance resources, the genetic basis of Verticillium wilt resistance in melon remains largely unexplored. The scarcity of characterized resistance genes has hindered the breeding of durable resistant cultivars, making the discovery and functional validation of novel resistance-associated loci an urgent priority for sustainable melon production.

Transcriptional reprogramming is a cornerstone of plant immune activation, and this process is tightly orchestrated by transcription factors and their regulatory co-factors. The VQ (valine-glutamine) motif-containing proteins constitute a family of plant-specific transcriptional regulators that function primarily by physically interacting with WRKY transcription factors to modulate their DNA-binding activity and transcriptional outputs [4,5]. Several VQ proteins have been functionally characterized in plant immunity across different species. In *Arabidopsis*, SIB1 and SIB2 act as co-activators of WRKY33 to potentiate defense against necrotrophic fungi [6]. In rice, the OsWRKY10-OsVQ8 module regulates diterpenoid phytoalexin biosynthesis through MAPK-mediated phosphorylation, conferring broad-spectrum resistance against blast and bacterial blight [7]. In tomato, SlVQ15 recruits SlWRKY30IIc and SlWRKY31 to integrate with the JA pathway, fine-tuning defense against root-knot nematodes [8,9].

Beyond their well-characterized WRKY interactions, emerging evidence indicates that VQ proteins also engage with other signaling components. For instance, VQ proteins have been shown to interact with MAP kinases, which modulate their stability and activity through phosphorylation, thereby linking VQ function to upstream signaling cascades [10,11]. Moreover, VQ proteins can associate with transcriptional regulators involved in the SA, JA, and ET pathways, forming complex regulatory networks that fine-tune hormonal outputs during defense [12,13,14].

In addition to their roles in immunity, VQ proteins are actively involved in the regulation of plant growth and developmental processes. Functional analyses using both knockout mutants and overexpression lines in *Arabidopsis* have revealed strong phenotypes in growth, development, and susceptibility to pathogen infection. For example, the *Arabidopsis* VQ18 and VQ26 proteins promote flowering by interacting with CONSTANS (CO) to enhance the transcriptional activation of FLOWERING LOCUS T (FT) [15], whereas VQ29 functions as a negative regulator of flowering transition, with its overexpression leading to delayed flowering [16]. VQ20 has been shown to modulate pollen development and function through interaction with WRKY2 and WRKY34 [5]. Beyond *Arabidopsis*, VQ proteins also participate in growth regulation in other species. In rice, *OsVQ1* knockout mutants exhibit a late-flowering phenotype under natural long-day conditions, while [17] OsVQ25 balances broad-spectrum disease resistance and plant growth through a hierarchical regulatory mechanism involving an E3 ligase and a transcription factor [18]. The dual involvement of VQ proteins in both immunity and development is consistent with the emerging view that transcriptional co-regulators often serve as nodes that integrate environmental stress signals with endogenous developmental programs [19]. This functional duality also predicts a trade-off between growth and defense when *VQ* genes are constitutively misexpressed, a phenomenon that we observed in our *CmVQ23*-overexpressing lines. Despite these advances, however, the functional roles of *VQ* genes in melon, particularly in the context of Verticillium wilt resistance have remained completely unexplored, representing a significant gap in our understanding of this important crop’s immune regulation.

In plants, defense against pathogens is orchestrated by a complex signaling network in which SA, JA, and ET serve as central hormonal hubs [20,21,22]. The SA pathway is typically associated with resistance against biotrophic and hemibiotrophic pathogens, whereas the JA/ET pathways are predominantly involved in defense against necrotrophic pathogens [23]. These pathways do not operate in isolation; rather, they exhibit extensive crosstalk that allows the plant to mount appropriate responses depending on the pathogen’s lifestyle [24]. Mitogen-activated protein kinase (MAPK) cascades act as key signaling modules that transduce extracellular stimuli into intracellular defense responses. Upon pathogen recognition, MAPK phosphorylation cascades activate downstream transcription factors and coordinate the accumulation of phytoalexins, ROS, and defense-related proteins [25]. Notably, MAPK-mediated phosphorylation has been reported to regulate the activity of VQ proteins, suggesting that VQ-containing modules function at the intersection of MAPK signaling and transcriptional reprogramming [10,11]. Understanding the interplay between VQ proteins, MAPK cascades, and hormonal signaling pathways is therefore essential for deciphering the regulatory architecture of plant immunity.

In a previous QTL mapping study using a recombinant inbred line population derived from a cross between resistant and susceptible melon accessions, we identified *CmVQ23* as a strong candidate resistance-associated gene. This gene encodes a typical VQ motif-containing protein, suggesting a potential role in the transcriptional regulation of defense responses. However, its biological function and the underlying mechanisms by which it may contribute to disease resistance have not been experimentally validated. In particular, whether CmVQ23 acts through WRKY-dependent or -independent mechanisms and how it interfaces with known defense signaling pathways remain open questions. This raises the central question of this study. Does CmVQ23 function as a positive regulator of Verticillium wilt resistance and, if so, through what molecular and physiological mechanisms?

To address this question, we adopted a heterologous expression strategy in *Arabidopsis*, taking advantage of its well-established genetic tools and robust pathogen infection system. We generated stable *CmVQ23*-overexpressing *Arabidopsis* lines and systematically evaluated their resistance to *V. dahliae* through comprehensive phenotyping, including disease severity scoring, fungal biomass quantification, and physiological parameter measurements. We further dissected the mechanistic basis of the observed resistance by examining ROS accumulation, hypersensitive response cell death, antioxidant enzyme activities, lignin deposition, photosynthetic performance, and the transcriptional activation of canonical defense marker genes. Through this multi-layered approach, we aim to elucidate whether CmVQ23 functions as a positive immune regulator and to unravel the integrated defense network it orchestrates. Our findings are expected to not only fill the knowledge gap regarding VQ protein function in melon but also provide a valuable genetic resource for improving Verticillium wilt resistance in this economically important crop.

## 2. Results

### 2.1. CmVQ23 Is Localized in the Nucleus

**Figure 1:**
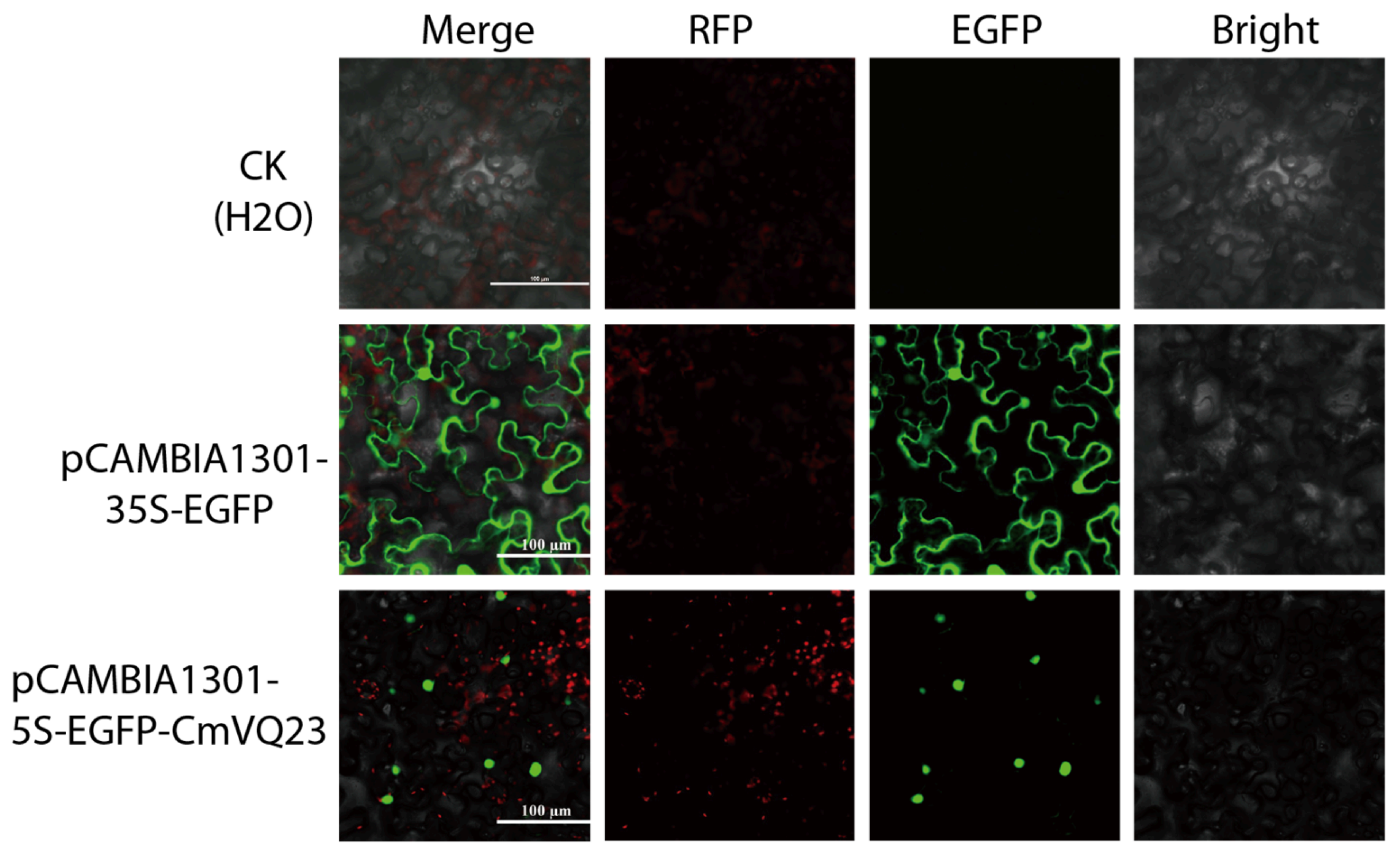
Subcellular localization of CmVQ23 in *Nicotiana benthamiana* leaves. RFP: chloroplast autofluorescence. Merge: overlay of bright field, eGFP, and RFP. Scale bars, 100 µm.

As a VQ motif-containing protein, CmVQ23 is postulated to function as a transcriptional co-factor, a role that typically requires nuclear localization. To test this hypothesis, we first examined the subcellular distribution of CmVQ23 using a transient expression assay in *Nicotiana benthamiana* leaves. The full-length *CmVQ23* coding sequence was fused to eGFP and placed under the control of the constitutive 35S promoter in the *pCAMBIA1301* vector. The resulting construct was transiently expressed in *N. benthamiana* leaves via agroinfiltration, and fluorescence signals were examined in lower epidermal cells at 48 h post-infiltration. In leaves infiltrated with the control vector expressing eGFP alone, fluorescence was evenly distributed throughout the cytoplasm, plasma membrane, and nucleus, indicating the non-specific localization of free eGFP. By contrast, the CmVQ23-eGFP fusion protein exhibited a strong nuclear signal with little or no fluorescence detected in the cytoplasm, suggesting specific nuclear localization (Figure 1). This nuclear localization is consistent with its predicted role as a transcriptional co-factor, as VQ proteins typically exert their regulatory functions in the nucleus by interacting with transcription factors to modulate their transcriptional activity [4,26,27]. Collectively, these results demonstrate that CmVQ23 is localized in the nucleus, supporting the hypothesis that it may function as a transcriptional co-factor involved in the melon immune response against *V. dahliae*.

To further characterize *CmVQ23* at the molecular level, we examined its gene structure and the presence of conserved functional motifs within the predicted protein sequence. The genomic organization of *CmVQ23* was analyzed by comparing its coding sequence (CDS) with the corresponding genomic sequence from the melon DHL92 v4 genome. As shown in Figure S2A, *CmVQ23* contains no introns, a feature that is consistent with most VQ protein-encoding genes in higher plants [28,29]. The full-length CmVQ23 protein comprises 143 amino acid residues and contains several conserved structural features relevant to its proposed function as a transcriptional co-factor (Figure S2B).

First, a canonical VQ motif (residues 39–64), containing the conserved core sequence VQEL (residues 56–60), was identified by NCBI CD-Search, confirming that CmVQ23 belongs to the VQ protein family. Second, a bipartite nuclear localization signal (NLS) was predicted at residues 20–41 (score = 8), which is consistent with its observed nuclear localization in our transient expression assay. Third, we identified two putative calmodulin (CaM)-binding IQ motifs at residues 38–53 and 101–116, as well as an additional CaM-binding site at residues 116–131, using the ELM (Eukaryotic Linear Motif) resource, suggesting that CmVQ23 may respond to calcium signaling during immune activation. Fourth, potential MAPK docking sites were predicted at residues 80–90 and 134–143 using ScanProsite, implying that CmVQ23 may be subject to phosphorylation-mediated regulation by MAPKs, a mechanism previously documented for several VQ proteins [10,11]. Collectively, these structural features position CmVQ23 as a VQ protein equipped with multiple functional domains that may facilitate its roles in transcriptional regulation, calcium sensing, and MAPK-mediated signal transduction during plant defense.

### 2.2. Generation and Characterization of CmVQ23 Transgenic Arabidopsis Lines

**Figure 2:**
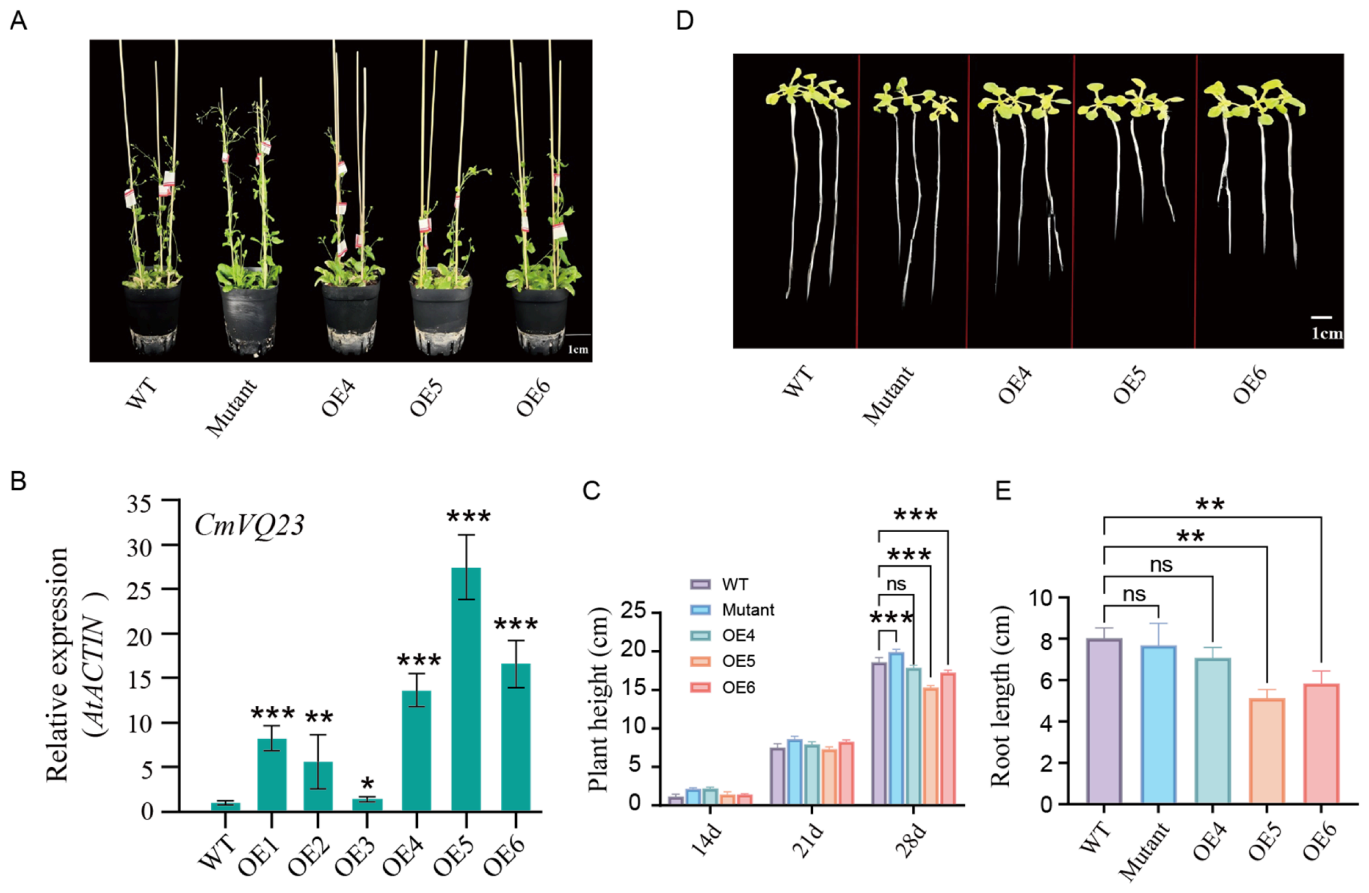
Overexpression of *CmVQ23* inhibits *Arabidopsis* growth. (**A**,**D**) Representative whole-plant and root growth images of WT, mutant, OE4, OE5, and OE6 plants grown under normal conditions for 20 days and 7 days, respectively. Scale bar = 1 cm. (**B**) RT-qPCR analysis of *CmVQ23* transcript level in overexpression lines relative to WT. Expression was normalized to the internal control *AtACTIN*. (**C**,**E**) Comparison of bolting length, root length, and plant height of WT, mutant, OE4, OE5, and OE6 during plant growth from 14, 21, and 28 days. Root length phenotypes at 5 days post-germination seedlings on 1/2 Murashige and Skoog (MS) medium were carefully transferred onto fresh vertical 1/2 MS medium and further cultured 3 days for observation. For each genotype, at least 30 individual plants were examined per biological replicate, and the entire experiment was repeated three times independently. ns, not significant; *, *p* < 0.05; **, *p* < 0.01; ***, *p* < 0.0001 (compared with WT).

To investigate the biological function of *CmVQ23*, we generated stable transgenic *Arabidopsis* lines overexpressing this melon gene under the control of the constitutive *35S* promoter via *Agrobacterium tumefaciens*-mediated transformation. Homozygous T3 lines were confirmed by qRT-PCR screening, and the transcript levels of *CmVQ23* were quantified by qRT-PCR using gene-specific primers (Table S1). Among six independent transgenic lines (OE1-OE6), three lines, OE4, OE5, and OE6, exhibited significantly higher expression levels, with OE5 showing the highest accumulation after normalization to the internal reference gene *AtACTIN*. The wild-type (WT) Col-0 plants showed no detectable expression of the transgene, as expected (Figure 2B). These three high-expressing lines were selected for all subsequent functional assays.

### 2.3. Overexpression of CmVQ23 Enhances Resistance to V. dahliae in Arabidopsis

**Figure 3:**
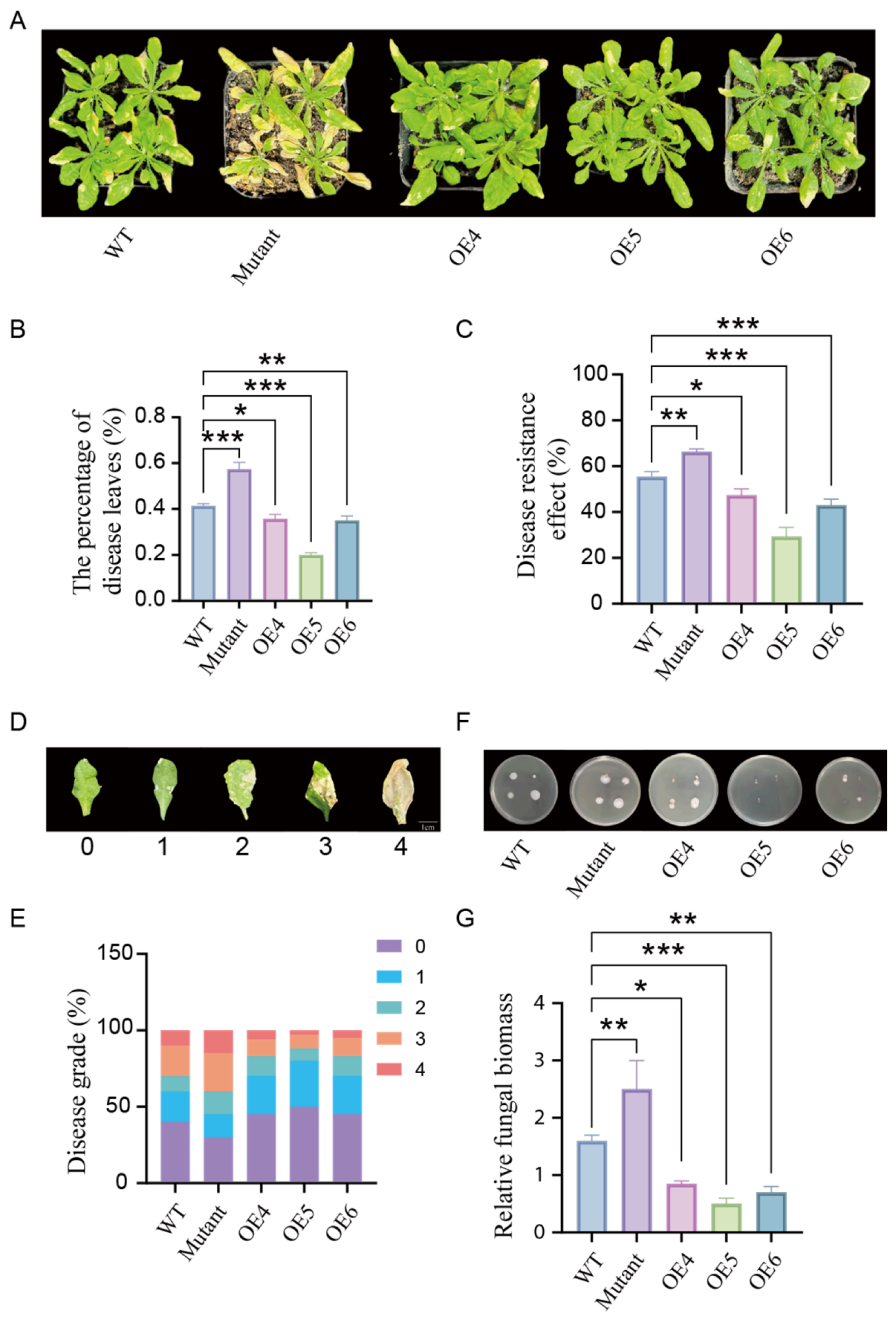
Ectopic overexpression of *CmVQ23* in *Arabidopsis* enhances resistance to *V. dahliae*. (**A**) Phenotypic comparison of *Arabidopsis* plants inoculated with *V. dahliae*. (**B**) Disease resistance efficiency of *Arabidopsis* plants post-VD991 inoculation. (**C**) Disease resistance effect. (**D**,**E**) Image and percentage of different disease levels in plants. Here, 0 to 4 represents disease severity score from low to high. (**F**) Fungal recovery assay. The stem segments of inoculated plants were placed on potato dextrose agar (PDA) medium, and photographs were obtained at 15 days after culture. (**G**) The fungal biomass of *Arabidopsis* seedlings inoculated with *V. dahliae*. The fungal biomass was determined by qPCR. Error bars indicate the SD with at least three biological replicates (n  ≥  3). *, **, and *** indicate statistical significance at the 0.05, 0.01, and 0.001 probability level using Student’s *t*-test.

To evaluate whether *CmVQ23* contributes to Verticillium wilt resistance, we inoculated WT, the three selected overexpression lines (OE4, OE5, and OE6), and a previously characterized mutant line (SALK_127478) with *V. dahliae* strain VD991 using a root drench method. A five-grade disease severity scale from 0 to 4 was established based on the leaf wilting phenotypes observed in WT plants at 15 days post-inoculation (dpi) (Figure 3D). Disease progression was monitored for 20 days, and the disease index as well as the proportion of diseased leaves were calculated for each genotype.

At 20 dpi, all three overexpression lines exhibited substantially lower disease indices and reduced proportions of diseased leaves compared with the WT (Figure 3A–C). The majority of transgenic plants fell into severity grades 0–1, with only mild chlorosis or no visible symptoms. In marked contrast, the mutant line showed a significantly higher disease index, with most plants displaying severe wilting and necrosis corresponding to grades 3–4 (Figure 3A,E).

To further corroborate these phenotypic observations, we performed a fungal re-isolation assay. Stem segments excised from inoculated plants at 15 dpi were surface-sterilized and placed on PDA medium. After 15 days of incubation, explants from the overexpression lines exhibited only minor or no fungal outgrowth, whereas those from the mutant line supported extensive mycelial proliferation, with markedly broader fungal growth zones compared with those observed in the overexpression lines (Figure 3F). Additionally, the fungal biomass in infected seedlings was quantified by qPCR using *V. dahliae* specific primers (Table S1). The overexpression lines harbored significantly lower fungal DNA levels than the WT, while the mutant line accumulated the highest fungal biomass (Figure 3G). Collectively, these results demonstrate that heterologous overexpression of *CmVQ23* significantly enhances *Arabidopsis* resistance to *V. dahliae* strain VD991.

### 2.4. Constitutive Overexpression of CmVQ23 Impairs Vegetative Growth in Arabidopsis

During phenotypic monitoring of the transgenic lines under normal growth conditions, we unexpectedly observed consistent alterations in vegetative development associated with *CmVQ23* overexpression. At 28 days after sowing, the OE4, OE5, and OE6 exhibited significantly reduced plant height compared with the WT, with the growth suppression becoming increasingly pronounced at later developmental stages (14, 21, and 28 days) (Figure 2A,C). In addition, these transgenic lines displayed delayed bolting relative to the WT, indicating a perturbation in the transition from vegetative to reproductive growth (Figure 2A).

The root development was also affected by *CmVQ23* overexpression. When grown on vertical 1/2 MS medium, the primary root length of the OE lines was significantly shorter than that of the WT at 7 days post-germination, whereas the mutant line showed no significant difference from the WT (Figure 2D,E). The overall root architecture of the overexpression lines appeared less branched, although this was not quantitatively assessed.

By contrast, the mutant line did not differ significantly from the WT in any of the measured shoot or root parameters, indicating that the observed growth phenotypes were specifically associated with *CmVQ23* overexpression rather than with genetic background variation. These observations suggest that the constitutive expression of this defense-related *VQ* gene incurs a detectable growth penalty.

### 2.5. CmVQ23 Promotes ROS Accumulation and Hypersensitive Response-Mediated Cell Death upon V. dahliae Infection

Given that a rapid and robust reactive oxygen species (ROS) burst is one of the earliest hallmarks of plant immune activation, we next examined whether CmVQ23-mediated resistance involves enhanced ROS production. To this end, we infiltrated leaves of the overexpression lines, the mutant, and WT with a conidial suspension of *V. dahliae* strain VD991 and performed histochemical staining at 12 h post-infiltration.

**Figure 4:**
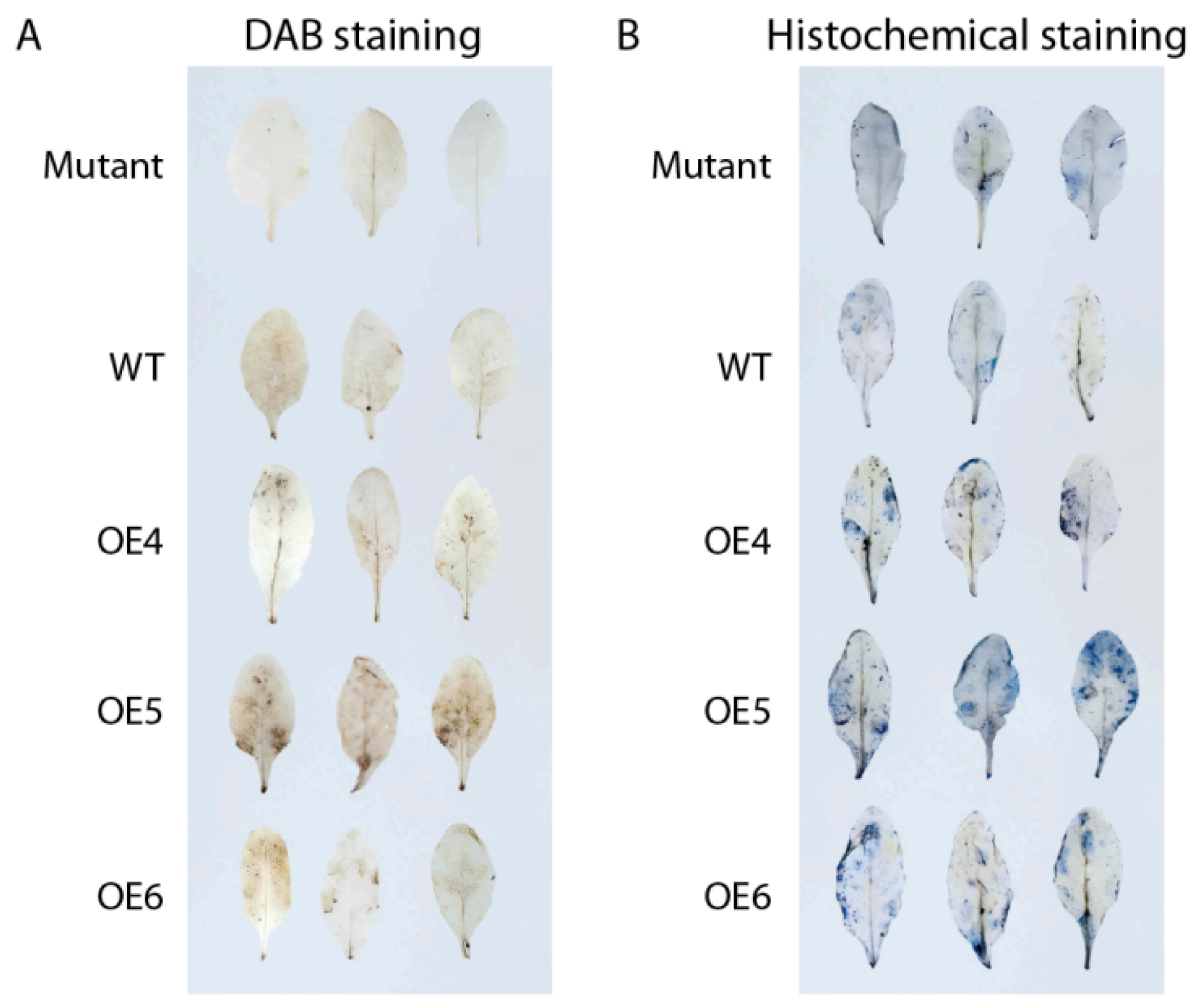
Accumulation of reactive oxygen species (ROS) in overexpression of *CmVQ23* in *Arabidopsis* plants after inoculation with *V.dahliae*. (**A**) 3,3′-diaminobenzidine (DAB) staining of *CmVQ23*-overexpression *Arabidopsis* leaves to detect the H_2_O_2_ accumulation at 12 h post-inoculation (hpi) with *V. dahliae*. (**B**) Trypan blue staining of *Arabidopsis* leaves at 12 hpi to detect cell death. Leaves were stained with trypan blue for 40 min and then subjected to microscopic observation. The extensive blue staining in the overexpression lines indicates a higher number of dead cells at the infection sites, reflecting HR-mediated programmed cell death. In contrast, the mutant line displayed considerably fewer dead cells, while the WT exhibited an intermediate level of staining.

We first assessed the hydrogen peroxide (H_2_O_2_) accumulation using 3,3′-diaminobenzidine (DAB) staining, which produces a brown polymerization product upon reaction with H_2_O_2_. The OE4, OE5, and OE6 exhibited intense brown staining localized around the infiltration sites, indicating markedly elevated H_2_O_2_ accumulation. In contrast, the WT showed only moderate staining, while the mutant line displayed minimal staining, reflecting a severely compromised oxidative burst (Figure 4A).

We next evaluated cell death at the infection sites using trypan blue staining, which penetrates only dead cells with compromised membranes, thereby reporting the occurrence of hypersensitive response (HR)-mediated programmed cell death. Consistent with the DAB staining results, the overexpression lines exhibited substantially darker and more extensive blue staining compared with the WT, indicating a higher number of dead cells at the infection sites. In marked contrast, the mutant line displayed considerably weaker staining, with only a few scattered blue cells (Figure 4B). Collectively, these results demonstrate that *CmVQ23* overexpression promotes not only ROS accumulation but also HR-mediated cell death at the sites of *V. dahliae* infection, suggesting that the enhanced resistance conferred by CmVQ23 involves the activation of programmed cell death as a defense mechanism to restrict pathogen spread.

The positive correlation between H_2_O_2_ accumulation and cell death suggests that the ROS burst triggered by *CmVQ23* overexpression likely acts as a signaling cue to initiate HR-associated programmed cell death, which serves to confine the biotrophic phase of *V. dahliae* and limit pathogen progression.

### 2.6. Overexpression of CmVQ23 Enhances Antioxidant Capacity and Promotes Lignin Deposition upon V. dahliae Infection

The enhanced ROS accumulation observed in the *CmVQ23* overexpression lines prompted us to investigate whether this response is accompanied by coordinated changes in the plant’s antioxidant defense system and structural reinforcement mechanisms. We therefore examined two interconnected layers of defense: (1) the enzymatic antioxidant capacity that regulates ROS homeostasis and (2) the activation of the phenylpropanoid pathway that leads to cell wall lignification.

**Figure 5:**
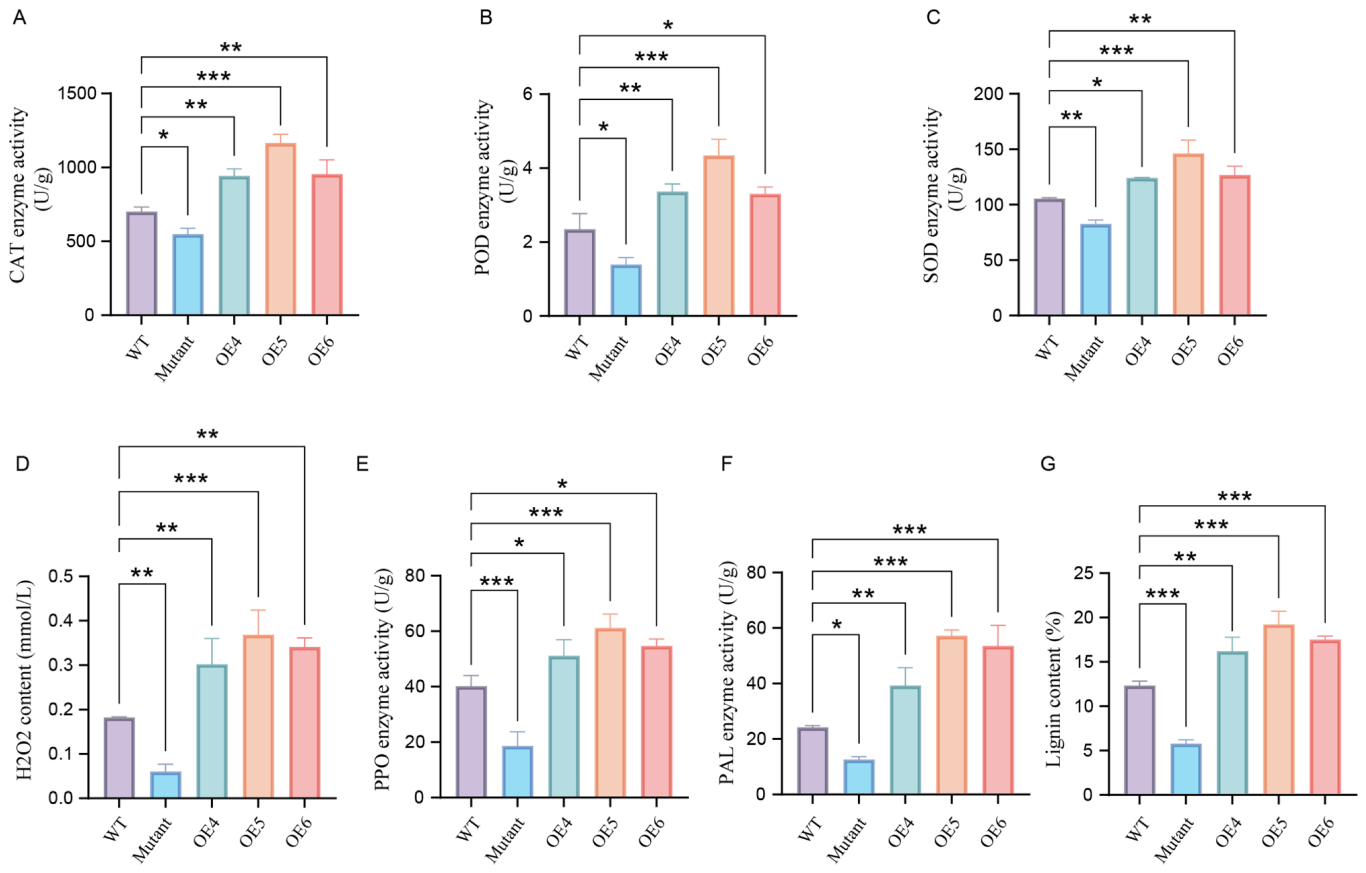
Overexpression of *CmVQ23* enhances antioxidant enzyme activities and increases lignin deposition upon *V. dahliae* infection. Activity of CAT (**A**), POD (**B**), SOD (**C**), H_2_O_2_ (**D**), PPO (**E**), PAL (**F**), and lignin (**G**) in transgenic and WT *Arabidopsis* treated with *V. dahliae*. Asterisks indicate significant differences compared with WT. *, *p* < 0.05; **, *p* < 0.01; ***, *p* < 0.0001 (Student’s *t*-test).

We first measured the activities of three key antioxidant enzymes, catalase (CAT), peroxidase (POD), and superoxide dismutase (SOD), alongside the H_2_O_2_ content in the same set of lines following *V. dahliae* inoculation. The overexpression lines exhibited significantly higher CAT, POD, and SOD activities compared with the WT, whereas the mutant line displayed the opposite trend (Figure 5A–C). Concurrently, the H_2_O_2_ content was also elevated in the overexpression lines relative to the WT (Figure 5D). While this may seem counterintuitive at first glance, the simultaneous increase in both ROS levels and antioxidant enzyme activities indicates that *CmVQ23* overexpression does not simply suppress oxidative stress but rather establishes a redox steady state at a higher baseline, one that sustains ROS-mediated signaling while preventing excessive oxidative damage. In this context, the enhanced antioxidant capacity serves a signal maintenance function, ensuring that the elevated ROS burst (Figure 4) is temporally and spatially controlled to propagate defense signals without causing uncontrolled cellular injury.

We next examined whether *CmVQ23* overexpression activates the phenylpropanoid pathway, a major metabolic route that supplies precursors for both lignin and diverse antimicrobial secondary metabolites. The activities of two key enzymes in this pathway, phenylalanine ammonia-lyase (PAL) and polyphenol oxidase (PPO), were significantly elevated in the overexpression lines compared with the WT, while the mutant showed substantially reduced activities (Figure 5E,F). Concomitantly, the lignin content was markedly higher in the overexpression lines and lower in the mutant (Figure 5G). These results demonstrate that *CmVQ23* overexpression potentiates the phenylpropanoid pathway, leading to enhanced lignin deposition that reinforces the plant’s structural barriers against fungal ingress. This lignin-mediated cell wall fortification constitutes a structural barrier function, physically restricting pathogen colonization and vascular spread.

Taken together, these findings reveal that CmVQ23 orchestrates a two-pronged defense strategy: on the one hand, it enhances the enzymatic antioxidant capacity to maintain ROS signaling within a controlled non-toxic range; on the other hand, it activates the phenylpropanoid pathway to reinforce cell wall integrity through lignin deposition. This coordinated regulation of both metabolic and structural defenses likely contributes substantially to the enhanced Verticillium wilt resistance observed in the *CmVQ23* overexpression lines.

### 2.7. Overexpression of CmVQ23 Maintains Higher Photosynthetic Capacity Under V. dahliae Stress

To determine whether *CmVQ23* influences photosynthetic performance under pathogen stress, we measured the chlorophyll fluorescence parameters in WT, mutant, and overexpression lines before and after inoculation with *V. dahliae* strain VD991. Four key parameters were assessed: Fv/Fm (maximum photochemical efficiency of PSII, reflecting the intrinsic health of the photosynthetic apparatus), Y(II) (effective quantum yield of PSII, indicating actual photochemical conversion efficiency), Y(NPQ) (quantum yield of regulated non-photochemical quenching, representing photoprotective energy dissipation), and Y(NO) (quantum yield of non-regulated energy dissipation, serving as a proxy for photodamage).

**Figure 6:**
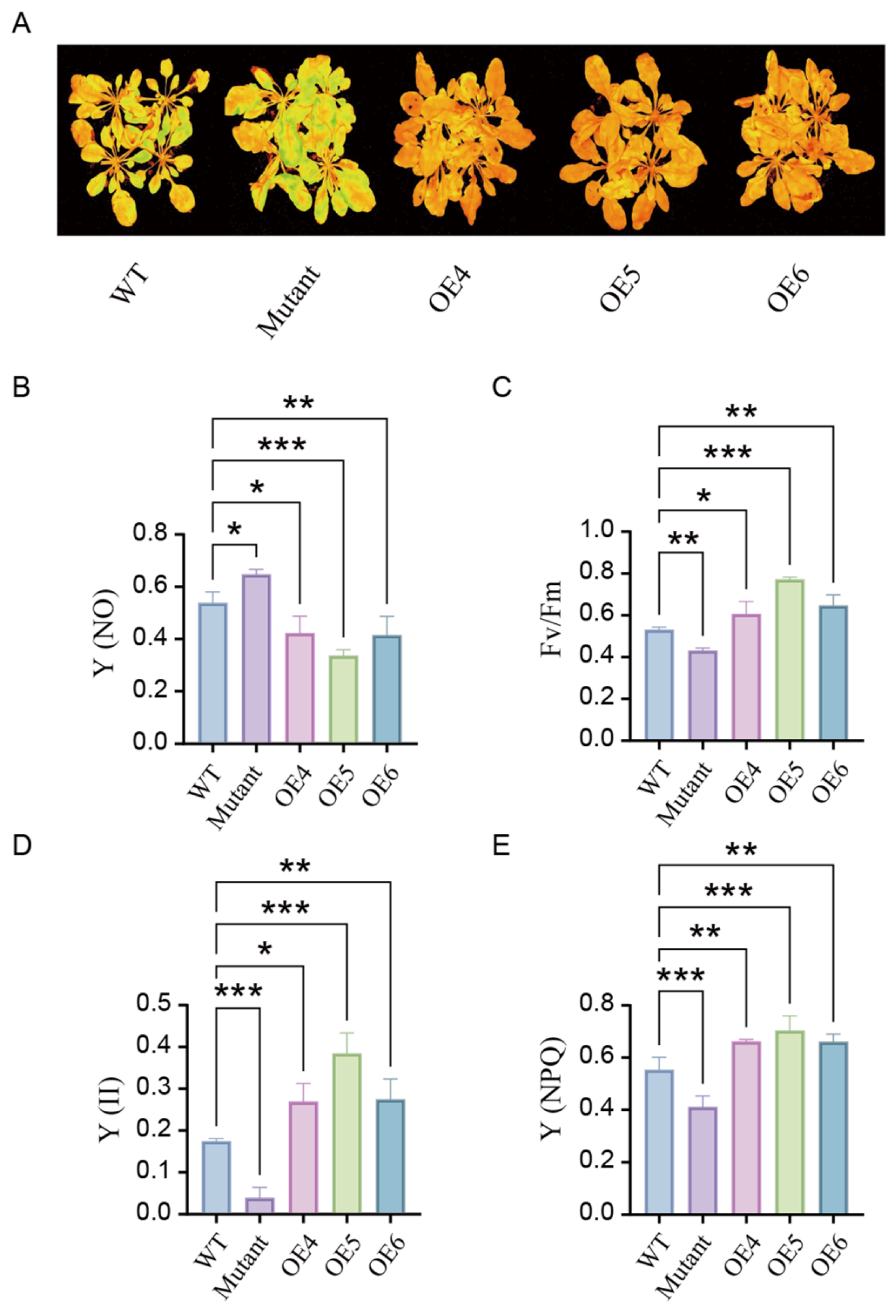
Overexpression of *CmVQ23* alleviates the inhibition of photosynthetic efficiency in *Arabidopsis* under *V. dahliae* stress. (**A**) Representative images showing disease symptoms and leaf wilting phenotypes at 15 dpi. (**B**) Quantum yield of non-regulated energy dissipation [Y(NO)]. (**C**) Maximum quantum yield of photosystem II (Fv/Fm). (**D**) Effective quantum yield of photosystem II [Y(II)]. (**E**) Quantum yield of regulated energy dissipation [Y(NPQ)]. All chlorophyll fluorescence parameters were recorded using a pulse-amplitude-modulation (PAM) fluorometer. Data are presented as means ± SD of three independent biological replicates (n = 3 per genotype). Asterisks indicate statistically significant differences compared with the WT group (*, *p* < 0.05; **, *p* < 0.01; ***, *p* < 0.001; Student’s *t*-test). The mutant line exhibits significantly reduced Fv/Fm, Y(NPQ) and Y(II), along with elevated Y(NO), indicating severe photoinhibition and enhanced thermal dissipation under pathogen attack. In contrast, all three overexpression lines (OE4-OE6) show increased Fv/Fm and Y(II) and decreased Y(NPQ), demonstrating that overexpression of *CmVQ23* protects PSII photochemical efficiency and mitigates energy dissipation, thereby contributing to improved *Arabidopsis* resistance to Verticillium wilt.

Upon *V. dahliae* infection, the OE4, OE5, and OE6 exhibited significantly higher Fv/Fm and Y(II) values compared with the WT, indicating that the photochemical efficiency and electron transport capacity were better preserved under pathogen challenge (Figure 6C,D). Concurrently, these lines showed elevated Y(NPQ), suggesting enhanced photoprotective thermal dissipation, and markedly reduced Y(NO), reflecting lower levels of photodamage relative to the WT (Figure 6B,E). In contrast, the mutant line displayed the opposite trend, with decreased Fv/Fm and Y(II), reduced Y(NPQ), and increased Y(NO), indicating severe photoinhibition and compromised PSII integrity under infection.

These results demonstrate that *CmVQ23* overexpression mitigates pathogen-induced photoinhibition and sustains photosynthetic capacity under *V. dahliae* stress. The preservation of photosynthetic efficiency is of particular significance, as it ensures continued energy supply and carbon skeleton production, which are essential to fuel the elevated metabolic demands of the activated defense responses.

### 2.8. Overexpression of CmVQ23 Activates Both SA- and JA/ET-Responsive Defense Genes upon V. dahliae Infection

The enhanced resistance and maintained photosynthetic capacity observed in the *CmVQ23* overexpression lines prompted us to examine whether this beneficial effect is associated with the activation of canonical defense signaling pathways. To this end, we quantified the transcript levels of well-established marker genes for SA-mediated and JA/ET-mediated defense responses in WT, mutant, and overexpression lines before and after *V. dahliae* inoculation.

**Figure 7:**
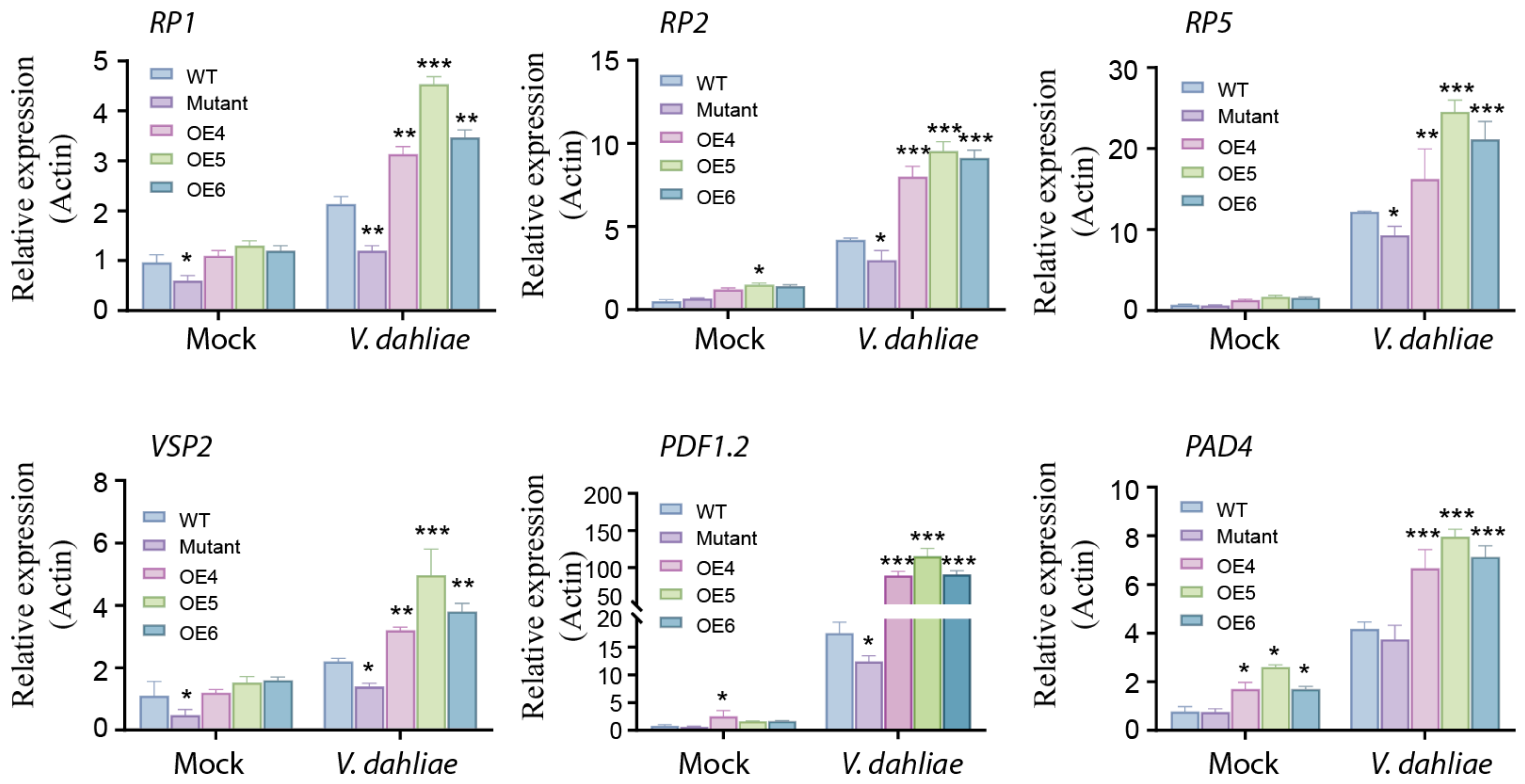
Overexpression of *CmVQ23* upregulates defense-related genes upon *V. dahliae* infection. Relative transcript levels of SA-responsive genes (*AtPR1*, *AtPR2*, *AtPR5*, and *AtPAD4*) and JA/ET-responsive genes (*AtPDF1.2* and *AtVSP2*) in WT, mutant, and overexpression lines of *Arabidopsis* under mock and *V. dahliae* inoculation conditions. Three-week-old seedlings were inoculated with *V. dahliae* strain VD991, and rosette leaves were harvested at 3 dpi for qRT-PCR analysis. Expression values were normalized to the internal control (*AtACTIN*) and are presented relative to the WT mock sample. Under mock conditions, no significant differences in gene expression were observed among all genotypes. Upon *V. dahliae* infection, the overexpression lines exhibited substantially higher transcript levels of all tested defense genes compared with WT, whereas the mutant displayed markedly reduced expression. Data represent three independent biological replicates. Asterisks indicate significant differences relative to WT (*, *p* < 0.05; **, *p* < 0.01; ***, *p* < 0.001; Student’s *t*-test).

We first examined the expression of four SA pathway marker genes: *AtPR1*, *AtPR2*, *AtPR5*, and *AtPAD4*. Under mock conditions, the transcript levels of these genes were comparable across all genotypes. Upon *V. dahliae* infection, the overexpression lines exhibited significantly elevated expression of all four SA-responsive genes compared with the WT, with *AtPR1* and *AtPR2* showing the most pronounced upregulation. In contrast, the mutant line displayed markedly reduced transcript levels for all four genes relative to the WT (Figure 7). These results indicate that *CmVQ23* overexpression potentiates the SA-dependent defense branch upon pathogen challenge.

We next assessed the expression of two JA/ET pathway marker genes, *AtPDF1.2* and *AtVSP2*. Similar to the SA pathway genes, no significant differences in transcript levels were observed among genotypes under mock conditions. Following *V. dahliae* inoculation, however, the overexpression lines showed substantially higher expression of both *AtPDF1.2* and *AtVSP2* compared with the WT, while the mutant line exhibited significantly reduced transcript levels (Figure 7). These findings demonstrate that *CmVQ23* overexpression also potentiates the JA/ET-dependent defense branch.

These results provide compelling evidence that CmVQ23 positively regulates both SA- and JA/ET-mediated defense signaling pathways in response to *V. dahliae* infection. Notably, the upregulation of both sets of marker genes in the overexpression lines indicates that *CmVQ23* does not bias the immune response toward a single hormonal pathway but rather acts as a broad-spectrum immune regulator that simultaneously engages both major defense signaling cascades. This dual activation may be particularly important for combating *V. dahliae*, a hemibiotrophic pathogen that likely requires coordinated SA and JA/ET signaling for effective resistance. The ability of CmVQ23 to concurrently amplify both defense branches distinguishes it from many known immune regulators that typically prioritize one pathway over the other.

## 3. Discussion

In this study, we functionally characterized CmVQ23, a melon VQ motif-containing protein, as a positive regulator of resistance against the vascular wilt pathogen *Verticillium dahliae*. Through heterologous overexpression in *Arabidopsis thaliana*, we demonstrated that *CmVQ23* confers enhanced tolerance by orchestrating a multifaceted defense response that includes ROS-triggered hypersensitive cell death, redox homeostasis maintenance, phenylpropanoid pathway activation, photosynthetic protection, and synergistic crosstalk between SA and JA/ET signaling. To our knowledge, this is the first report of a functionally characterized VQ protein involved in disease resistance in melon, and it provides a valuable entry point for understanding the regulatory architecture of immunity in this important crop species.

### 3.1. Comparison with Other VQ Proteins Reveals Both Conserved and Lineage-Specific Features

The VQ family has emerged as a class of important transcriptional co-regulators in plant immunity across diverse species, yet their functional modes exhibit considerable variation. In agreement with the established roles of VQ proteins in defense, our finding that CmVQ23 positively regulates resistance aligns with the positive regulatory functions reported for Arabidopsis SIB1/SIB2 (which activate camalexin biosynthesis via WRKY33 against necrotrophic fungi) [30], rice OsVQ8 (which partners with OsWRKY10 to orchestrate diterpenoid phytoalexin production) [7], and tomato SlVQ15 (which fine-tunes defense against root-knot nematodes through SlWRKY30IIc) [9]. However, CmVQ23 displays several distinctive properties that set it apart from these known examples.

First, unlike SlVQ15, which primarily operates through the JA pathway, CmVQ23 concurrently activates both SA- and JA/ET-responsive marker genes (Figure 7), indicating a broader regulatory scope. This dual engagement is particularly striking because SA and JA/ET pathways are often mutually antagonistic in many plant–pathogen interactions [31,32]. The ability to integrate both hormonal branches may be an adaptation to the hemibiotrophic lifestyle of *V. dahliae*, which requires both biotrophic-phase and necrotrophic-phase defenses for optimal resistance.

Second, while apple MdVQ37 and pumpkin CpVQ30 have been shown to act as negative regulators of resistance by suppressing ROS accumulation or reducing antioxidant enzyme activities [33,34,35], CmVQ23 functions as a positive regulator that promotes both ROS burst and antioxidant enzyme activities (CAT, POD, SOD) simultaneously (Figure 5A–D). This seemingly paradoxical co-elevation establishes a new redox steady state at an elevated set point, as discussed below, and contrasts sharply with the unbridled oxidative stress associated with susceptibility in those negative-regulator systems.

Third, *CmVQ23* overexpression maintains higher photosynthetic efficiency (Fv/Fm, Y(II), and Y(NPQ)) upon infection (Figure 6B–E), a feature not previously reported for any VQ protein. This physiological protection likely provides the metabolic energy and carbon skeletons required for sustained immune outputs, representing a novel layer of VQ-mediated defense that goes beyond the canonical transcriptional regulation.

### 3.2. A Multilayered Defense Network Orchestrated by CmVQ23

The resistance phenotype conferred by *CmVQ23* is underpinned by at least four interconnected layers that operate at both cellular and systemic levels. At the early response phase, overexpression triggers rapid H_2_O_2_ accumulation and hypersensitive cell death (Figure 4), providing both direct antimicrobial activity and a secondary signaling hub that amplifies downstream defenses [36,37]. This positive regulation of the ROS–HR axis distinguishes CmVQ23 from negative regulators such as apple MdVQ37 and pumpkin CpVQ30, which dampen this response. Concomitantly, the elevated antioxidant enzyme activities (CAT, POD, and SOD) prevent uncontrolled cellular damage while sustaining sufficient ROS signaling for immune activation (Figure 5A–D). In parallel, activation of PAL and PPO, together with increased lignin deposition (Figure 5E–G), reinforces cell wall structural barriers that physically impede fungal hyphal progression through the xylem, a particularly relevant defense against vascular pathogens. Intriguingly, the cotton R2R3-MYB transcription factor GhMYB315, which associates with GhVQ28 to regulate phenylpropanoid metabolism [38], suggests that VQ-mediated lignin reinforcement may represent a conserved defense strategy against *V. dahliae* across different plant species, although whether CmVQ23 similarly engages MYB partners or acts exclusively through WRKY transcription factors remains to be determined.

Beyond these local cellular defenses, *CmVQ23* overexpression also confers systemic physiological protection and coordinates broad-spectrum hormonal signaling. Chlorophyll fluorescence analysis revealed that transgenic lines maintained higher photosynthetic efficiency (Fv/Fm, Y(II), and Y(NPQ)) upon infection (Figure 6B–E), ensuring sustained energy and carbon supply for the elevated metabolic demands of defense, including phytoalexin and lignin biosynthesis [39]. This preservation of photosynthetic performance under pathogen challenge represents a previously unrecognized dimension of VQ-mediated immunity, expanding the conventional view of VQ proteins as mere transcriptional co-factors. At the hormonal level, *CmVQ23* simultaneously upregulates both SA- and JA/ET-responsive marker genes upon *V. dahliae* infection (Figure 7), indicating that it functions as a signal integrator that coordinates multiple hormonal outputs. This dual activation is particularly noteworthy, because SA and JA/ET pathways are often mutually antagonistic in many plant–pathogen interactions [31,32]. The ability to engage both signaling branches simultaneously confers a broader regulatory scope than that of tomato SlVQ15, which primarily operates through the JA pathway [9], and may be essential for countering the complex infection cycle of *V. dahliae*, a hemibiotrophic fungus that requires both biotrophic- and necrotrophic-phase defenses for optimal resistance. Collectively, these coordinated layers suggest that CmVQ23 occupies a relatively high hierarchical position in the defense signaling network, possibly functioning as a master switch that fine-tunes both metabolic and structural defenses while maintaining overall plant fitness during infection.

### 3.3. Limitations and Future Perspectives

Despite the significant insights gained, several important limitations of the current study should be acknowledged and addressed in future work. First, the specific WRKY transcription factor or factors that physically interact with CmVQ23 have yet to be identified. Although nuclear localization and the conserved VQ motif strongly suggest WRKY binding, the partner or partners mediating the regulatory function of CmVQ23 may be lineage-specific and may not have obvious orthologs in *Arabidopsis*. Future yeast two-hybrid screening or co-immunoprecipitation coupled with mass spectrometry (Co-IP/MS) using melon cDNA libraries will be essential to identify its native interaction partners. Beyond WRKYs, it is also plausible that CmVQ23 may engage with other classes of transcription factors, such as MYB, NAC, or ERF family members, given the emerging evidence that VQ proteins can interact with a broader spectrum of regulatory proteins, depending on the cellular context [38,40]. Second, while our heterologous expression in *Arabidopsis* has provided valuable functional insights, the ultimate validation of CmVQ23’s role in Verticillium wilt resistance must be performed in its native host, melon, through CRISPR/Cas9-mediated gene editing or natural allele mining in diverse germplasm collections. Third, the detailed molecular mechanisms by which CmVQ23 coordinates ROS signaling, phenylpropanoid metabolism, and phytohormone crosstalk require further dissection, potentially through transcriptomic and phosphoproteomic analyses. Fourth, our findings position *CmVQ23* as a candidate allelic variant potentially contributing to the resistance phenotype of MR1 melon. In susceptible genotypes, the corresponding allele may exhibit reduced transcript abundance under pathogen challenge, carry nonsynonymous mutations that compromise protein stability or nuclear localization, or show altered affinity for its cognate WRKY transcription factors, thereby failing to mount the full multilayered defense response described here. It is also plausible that promoter polymorphisms affect the pathogen-inducible expression of the gene, leading to insufficient activation of ROS, phenylpropanoid, and hormonal signals upon *V. dahliae* infection. To test these hypotheses, future work should compare the allelic sequences, expression kinetics, and protein–protein interaction capacities of CmVQ23 between resistant (MR1) and susceptible melon lines, preferably using CRISPR/Cas9-mediated allele swap or natural variation studies. Addressing these questions will not only deepen our understanding of VQ protein function in melon immunity but also facilitate the translation of this knowledge into practical strategies for Verticillium wilt resistance breeding.

## 4. Materials and Methods

### 4.1. Plant Materials and Growth

*Arabidopsis thaliana* accession Col-0 was used as the wild-type control. For surface sterilization, seeds were first immersed in 75% ethanol for 5 min, followed by treatment with 2% sodium hypochlorite for 10 min with gentle shaking. The seeds were then rinsed five times with sterile distilled water. After sterilization, seeds were stratified at 4 °C in darkness for 3 days and subsequently sown on half-strength Murashige and Skoog (1/2 MS) medium containing 1% (*w*/*v*) sucrose and 0.7% (*w*/*v*) agar. Seven-day-old seedlings were transplanted into a soil mixture of vermiculite, peat moss, and compost–topsoil blend at a 1:1:1 ratio (*v*/*v*). Unless otherwise stated, plate-grown seedlings and soil-grown plants were cultivated under a long-day photoperiod (16 h light/8 h dark, 125 µmol m^−2^ s^−1^) with day/night temperatures of 21 °C and 19 °C, respectively.

### 4.2. Subcellular Localization of CmVQ23

cDNA from the resistant parent MR1 was used as a template to amplify the *CmVQ23* gene using gene-specific primers (Table S1) that contained homologous arms for recombination. The resulting PCR products were inserted into the binary vector pCAMBIA1301-35S-EGFP via homologous recombination, yielding C-terminal EGFP fusion constructs driven by the *CaMV 35S* promoter. Each construct was separately transformed into *A. tumefaciens* strain GV3101.

For subcellular localization assays, *N. benthamiana* plants at the five- to six-leaf stage were infiltrated with Agrobacterium cultures harboring each construct or the empty vector (negative control) on the abaxial side of the leaves. After infiltration, the plants were incubated under low-light conditions for two days. Leaf discs were then excised from the infiltrated zones and observed under a laser scanning confocal microscope. eGFP was excited at 488 nm with emission collected at 510 nm, whereas chloroplast autofluorescence was detected at an excitation wavelength of 645 nm. Images were captured for each sample.

### 4.3. Agrobacterium-Mediated Genetic Transformation of A. thaliana

For genetic transformation, the binary vector pCAMBIA3301 was used. Plasmid propagation was performed in *E. coli* DH5α, and *A. tumefaciens* strain GV3101 was employed for plant transformation. The gene *CmVQ23* gene was cloned into the pCAMBIA3301 vector between the *SacI* and *PstI* restriction sites. After sequence verification, the recombinant plasmid was transformed into *A. tumefaciens* GV3101. *A. thaliana* plants were transformed via the floral dip method. To confirm transgene integration, transgenic plants were screened by PCR with gene-specific primers (Table S1), and positive lines were selected for further analysis.

To identify the *Arabidopsis* ortholog of the melon gene *MELO3C009755*, BLASTP (NCBI, https://blast.ncbi.nlm.nih.gov) sequence alignment was performed using the melon reference genome Melon (DHL92) v4, available at the Cucurbit Genomics Database (http://cucurbitgenomics.org/). The search against the Arabidopsis TAIR10 protein database identified AT3G56710 as the closest homolog. According to the TAIR database, AT3G56710 encodes SIB1 (Sigma factor binding protein 1), which is also designated as AtVQ23 (or VQ23) [41]. Based on this orthologous relationship, we have renamed the melon gene *MELO3C009755* as *CmVQ23* to reflect its homology to *AtVQ23*. To further validate this assignment, a systematic genome-wide identification of VQ motif-containing proteins was performed in melon, and a phylogenetic tree was constructed together with all *Arabidopsis* VQ proteins. The phylogenetic analysis confirmed that CmVQ23 and AT3G56710 (AtVQ23/SIB1) cluster within the same evolutionary clade, supporting their close orthologous relationship (Figure S1).

### 4.4. RNA Isolation and qPCR Analysis

Total RNA was isolated from leaves using the Plant Total RNA Isolation Kit Plus (Vazyme Biotech Co., Ltd., Nanjing, China), according to the manufacturer’s instructions. Three independent biological samples were prepared. First-strand cDNA was synthesized using Master Premix RT Easy^TM^ (Tiangen Biotech Co., Ltd., Beijing, China), following the manufacturer’s instructions. qRT-PCR was performed on an ABI QuantStudio 3 system (abm, Waltham, MA, USA) in 20 μL reactions containing 10 μL Real Time PCR EasyTM-SYBR Green I (Yugong Biotech Co., Ltd., Nanjing, China), 2 μL cDNA template, 0.5 μL each of forward/reverse primers, and 7 μL nuclease-free water. The thermal cycling protocol comprised initial denaturation at 95 °C for 3 min, followed by 40 cycles of 95 °C for 15 s and 60 °C for 1 min. *AtActin* served as the internal reference genes, with the relative gene expression calculated using the comparative 2^−ΔΔCt^ method [42]. Primers for all qPCR reactions are listed in Table S1.

### 4.5. Activation of V. dahliae and Fungal Inoculation

*V. dahliae* strain VD991 was activated on PDA medium by streaking and incubated at 28 °C for 4 days. Subsequently, agar plugs containing mycelia were transferred to PDA liquid medium and cultured at 28 °C on a rotary shaker at 200 rpm for 4–6 days. The activated fungal culture was filtered through three layers of sterile gauze to remove mycelial debris. The spore concentration was determined using a hemocytometer and adjusted to 1 × 10^7^ colony-forming units (cfu)/mL with sterile distilled water.

For inoculation, a root drench method was employed. Each *Arabidopsis* plant received 15 mL of the spore suspension, applied in two separate injections: first, 10 mL was injected into the soil around the roots, followed by another 5 mL on the next day. To maintain humidity, the plants were covered with plastic wrap. The experiment was performed with three independent biological replicates, each comprising at least 10 plants per replicate.

### 4.6. Disease Assessment

The disease severity was evaluated based on the percentage of diseased leaves and the disease index. The percentage of diseased leaves was calculated as the number of diseased leaves divided by the total number of leaves examined, multiplied by 100%. For the disease index, the severity of each plant was classified into six grades (0 to 4) according to the following criteria: grade 0, healthy growth with no chlorosis, wilting, or lesions; grade 1, less than 25% of leaves showing yellowing, wilting, or lesions; grade 2, 25–50% of leaves showing yellowing, wilting, or lesions; grade 3, 50–75% of leaves showing yellowing, wilting, or lesions with a few leaves dried; grade 4, more than 75% of leaves wilted or detached or the whole plant dead. The disease index was then computed as the sum of (number of plants in each grade multiplied by the grade value) divided by the total number of plants examined multiplied by 4, and the result was expressed as a percentage.

### 4.7. Measurement of Physiological and Biochemical Parameters

The contents of hydrogen peroxide (H_2_O_2_), peroxidase (POD), superoxide dismutase (SOD), catalase (CAT), PAL, lignin, and PPO were measured using commercial assay kits following the manufacturer’s instructions (Beijing Solarbio Science & Technology Co., Ltd., Beijing, China).

### 4.8. Measurement of Photosynthetic Capacity

Chlorophyll fluorescence parameters were measured using an IMAGING-PAM chlorophyll fluorometer (Heinz Walz GmbH, Effeltrich, Germany). The third and fourth fully expanded mature leaves of four-week-old plants from different lines were selected for measurement before and after stress treatment. The following parameters were recorded: maximum photochemical efficiency of photosystem II (Fv/Fm), actual photochemical efficiency of photosystem II (Y(II)), quantum yield of non-regulated energy dissipation (Y(NO)), and non-photochemical quenching (NPQ).

### 4.9. Stem Segment Fungal Re-Isolation Assay

Stem segments were collected from each line at 15 dpi with *V. dahliae* strain VD991 and cut into approximately 1 cm pieces. The segments were surface-sterilized with 75% ethanol for 1 min, rinsed three times with sterile distilled water, and blotted dry on sterile filter paper. The sterilized segments were then placed on PDA medium for cultivation. Each Petri dish contained six *Arabidopsis* stem segments. For each line, three replicate dishes were used, and the entire experiment was repeated three times independently.

### 4.10. DAB Staining and Trypan Blue Staining

For DAB staining, a 1 mg/mL DAB solution was prepared and stored at 4 °C in the dark. Leaves from the same position of plants before and after treatment were collected and immersed in the DAB staining solution, followed by incubation at 37 °C in the dark for 8–12 h. After staining, the leaves were boiled in 95% ethanol for decolorization and then photographed. At least 10 leaves were used for each treatment.

For trypan blue staining, the staining solution was prepared by dissolving 5 mg of trypan blue in a mixture containing 5 mL lactic acid, 5 mL phenol, 5 mL glycerol, and 5 mL sterile water. Leaves were soaked in this solution for 2 h. After staining, the solution was removed, and the leaves were gently washed with 95% ethanol. The ethanol was then replaced with fresh 95% ethanol, and the leaves were boiled for 5 min. This ethanol boiling step was repeated until the chlorophyll was completely removed from the leaves. Finally, the leaves were photographed. At least 10 leaves were examined per treatment.

### 4.11. Statistical Analysis

All experiments were performed with three or more independent replicates. Data are presented as the means ± standard error (SE). Data processing was carried out using Microsoft Excel 2022, and statistical analysis was performed using GraphPad Prism 9.5.0. Differences between groups were analyzed by one-way analysis of variance (ANOVA) as appropriate, and graphs were generated using the same software.

## 5. Conclusions

In this study, we functionally characterized *CmVQ23*, a VQ motif-containing gene previously identified as a candidate resistance-associated locus through QTL mapping in melon, by heterologous expression in *A. thaliana*. Our findings demonstrate that CmVQ23 acts as a positive regulator of plant immunity against *V. dahliae*. Upon pathogen challenge, *CmVQ23* overexpression promotes ROS accumulation and HR-mediated cell death at infection sites, while simultaneously enhancing antioxidant enzyme activities to maintain ROS signaling within a controlled range. Concurrently, it activates the phenylpropanoid pathway, leading to increased lignin deposition that reinforces structural barriers against fungal ingress, and preserves photosynthetic efficiency under pathogen stress. At the transcriptional level, CmVQ23 upregulates both SA- and JA/ET-responsive marker genes upon infection, indicating that it orchestrates defense signaling through synergistic activation of multiple phytohormone pathways. These results establish CmVQ23 as a multifaceted positive regulator that integrates ROS/HR-mediated defense, antioxidant reinforcement, cell wall fortification, and phytohormone signaling crosstalk.

## Data Availability

The original contributions presented in this study are included in the article/Supplementary Material. Further inquiries can be directed to the corresponding author.

## Supplementary Materials

The following supporting information can be downloaded at https://www.mdpi.com/article/10.3390/plants15152283/s1, Table S1. Primers were used for amplification, subcelluar localization, gene expression validation, and fungal biomass. Figure S1. Phylogenetic relationship between melon and Arabidopsis VQ family genes (See Ref. [43]). Figure S2. Gene and protein structures of CmVQ23.

## Abbreviations

The following abbreviations are used in this manuscript:

ROS	Reactive Oxygen Species
HR	Hypersensitive Response
PAL	Phenylalanine Ammonia-Lyase
PPO	Polyphenol Oxidase
SA	Salicylic Acid
JA	Jasmonic Acid
ET	Ethylene
MS	Murashige and Skoog
DPI	Days Post-Inoculation
PDA	Potato Dextrose Agar
DAB	3,3′-DiAminoBenzidine
CFU	Colony-Forming Unit
