# Supplementary material for: Heterologous Expression of the Melon CmVQ23 Positively Regulates Resistance to Verticillium dahliae in Arabidopsis
Source: Plants (Basel). 2026 Jul 26;15(15):2283. doi: 10.3390/plants15152283 (PMC13468026; doi:10.3390/plants15152283)
Supplement: Supplementary file 1 [file plants-15-02283-s001.zip › plants-4452236-supplementary.pdf]

Table S1. Primers were used for amplification, subcelluar localization, gene expression validation, and fungal biomass.

Primer sequences (5′-3′)	Primer sequence (5′-3′)	Purpose of primers
1301-*CmVQ23*-F	AGAACACGGGGGACGAGCTCGGTACCATGGATCGAACTTTACTGGTAAGTG	Primers for subcellular localization
1301-*CmVQ23*-R	CCCTTGCTCACCATGTCGACTCTAGAAACCAAAGGATTGATTAAAGAT	Primers for subcellular localization
3301-*CmVQ23*-F	GGGACGAGCTCGGTACCCGGGGATCCATGGATCGAACTTTACTGGTAAGTG	Primers for subcellular localization
3301-*CmVQ23*-R	CATGGAAGATCTTCGTCGACTCTAGACTAAACCAAAGGATTGATTAAAGAT	Primers for subcellular localization
qRT-*CmVQ23*-F	CGAAGAGAATAAGCAACCCCT	Primers for *CmVQ23* expression
qRT-CmVQ23-R	TCGCACGACGCTGGATAGAAC	Primers for *CmVQ23* expression
Vd991-F	CGTTTCCCGTTACTCTTCT	Quantification of fungal biomass
Vd991-R	GGATTTCGGCCCAGAAACT	Quantification of fungal biomass
qRT-PCR-*AtPR1*-F	TCGTCTTTGTAGCTCTTGTAGGTG	Real-time quantification
qRT-PCR-*AtPR1*-R	TTCATTAGTATGGCTTCTCGTTCA	Real-time quantification
qRT-PCR-*AtPR2*-F	CCACCAATGTTGATGATTCTTCT	Real-time quantification
qRT-PCR-*AtPR2*-R	ATACTCATCCCTGAACCTTCCTT	Real-time quantification
qRT-PCR-*AtPDF1.2*-F	TCACCCTTATCTTCGCTGCTC	Real-time quantification
qRT-PCR-*AtPDF1.2*-R	ATGTCCCACTTGGCTTCTCG	Real-time quantification
qRT-PCR-*AtPR5*-F	AGGATTTGAATTGACTCCAGGT	Real-time quantification
qRT-PCR-*AtPR5*-R	CCATCGCCTACTAGAGTGAATT	Real-time quantification
qRT-PCR-*AtVSP2*-F	TGTGAACAGGCAGATGAACC	Real-time quantification
qRT-PCR-*AtVSP2*-R	GCGATACCGATCTCGTCAA	Real-time quantification

qRT-PCR-*AtPAD4*-F	CTATTCTGAGGTCAGCTGAG	Real-time quantification
qRT-PCR-*AtPAD4*-R	CGCATAACTCTCGAATGGAAC	Real-time quantification

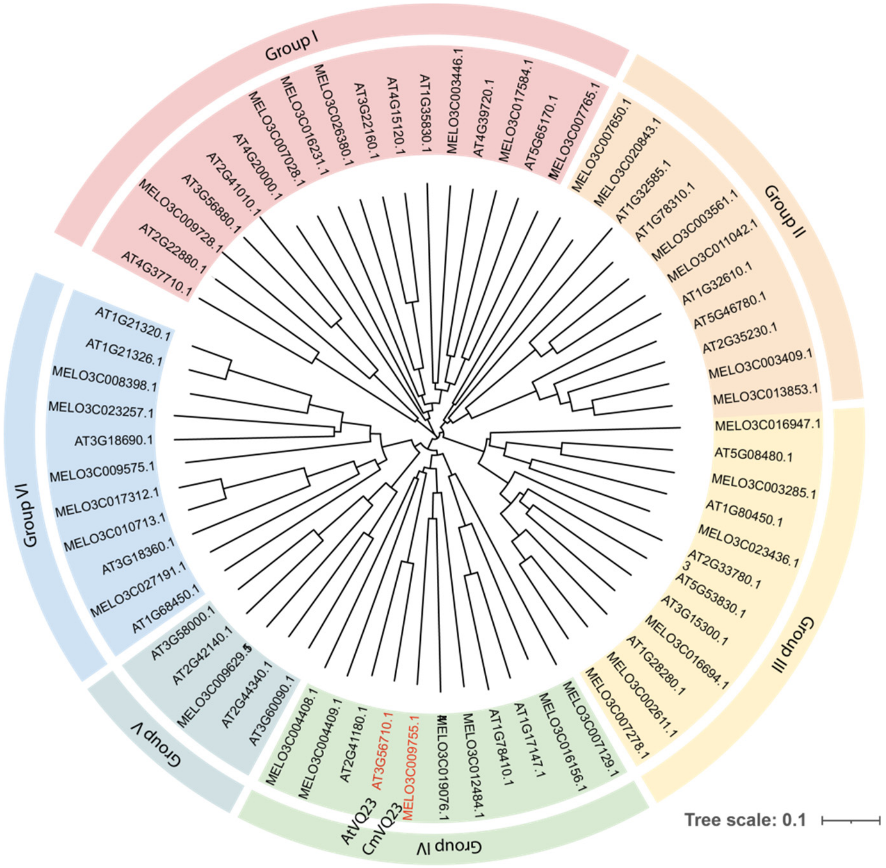

Figure S1. Phylogenetic relationship between melon and Arabidopsis VQ family genes. The phylogenetic tree was constructed using MEGA11 with the neighbor-joining (NJ) method and 1,000 bootstrap replicates, based on the full-length amino acid sequences of VQ proteins from *Cucumis melo* (DHL92 v4 genome) and *Arabidopsis thaliana* (TAIR10). The tree was visualized and annotated using iTOL v6 (https://itol.embl.de) [1]. Conserved motif structures of melon VQ proteins were analyzed using the online MEME software (http://www.omicsclass.com/article/67), with the maximum number of motifs set to 10, minimum motif width to 6, and maximum motif width to 50. Different colored boxes represent distinct conserved motifs, and their relative positions within each protein are shown proportionally. The scale bar indicates the evolutionary distance (substitutions per site). Bootstrap values (≥50%) are shown at the nodes. The melon protein MELO3C009755 (designated as CmVQ23 in this study) and its *Arabidopsis* ortholog AT3G56710 (AtVQ23/SIB1) are marked with asterisks.

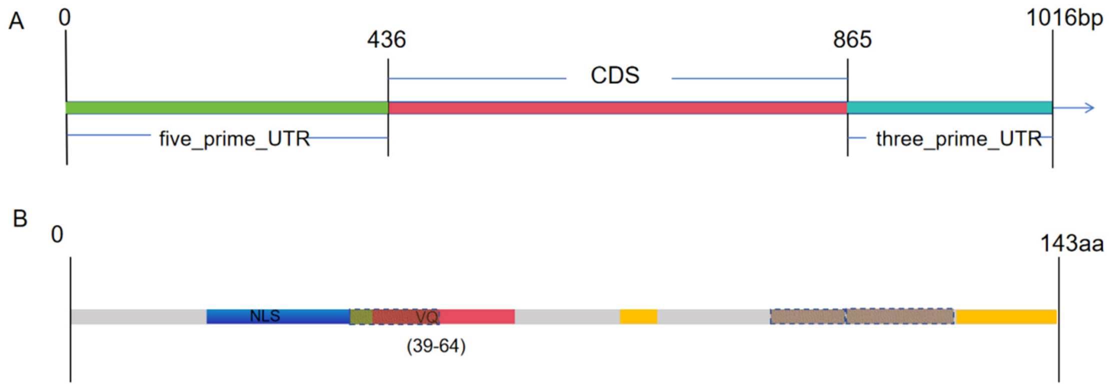

Figure S2. Gene and protein structures of CmVQ23. A: Gene structure of *CmVQ23*; B: Schematic representation of the CmVQ23 protein. The protein is 143 amino acids in length. Only motifs with well-documented functions in transcriptional regulation, nuclear import, calcium signaling, and MAPK-mediated phosphorylation are shown. The VQ motif (39-64aa,red) containing the VQEL core (56-60aa) was identified by NCBI CD-Search. A bipartite nuclear localization signal (NLS, residues 20-41, score = 8, blue) supports its nuclear localization. Putative calmodulin-binding IQ motifs (38-53, 101-116, 116-131, brown) and MAPK-docking sites (80-90, 134-143, orange) were identified by ELM and ScanProsite, respectively.

1. Letunic I, Bork P. Interactive Tree of Life (iTOL) v6: recent updates to the phylogenetic tree display and annotation tool. Nucleic Acids Res. 2024;52(W1):W78-w82.
